# Enhanced Electrochemical Properties and OER Performances by Cu Substitution in NiCo_2_O_4_ Spinel Structure

**DOI:** 10.3390/nano10091727

**Published:** 2020-08-31

**Authors:** Hyerim Park, Byung Hyun Park, Jaeyoung Choi, Seyeon Kim, Taesung Kim, Young-Sang Youn, Namgyu Son, Jae Hong Kim, Misook Kang

**Affiliations:** 1 Department of Chemistry, College of Natural Sciences, Yeungnam University, Gyeongsan 38541, Gyeongbuk, Korea; ilkilhs1329@ynu.ac.kr (H.P.); pbh0326@ynu.ac.kr (B.H.P.); hty555@naver.com (J.C.); ksy4219@naver.com (S.K.); ktsart@naver.com (T.K.); ysyoun@yu.ac.kr (Y.-S.Y.); 2 School of Chemical Engineering, College of Engineering, Yeungnam University, Gyeongsan 38541, Gyeongbuk, Korea; jaehkim@ynu.ac.kr

**Keywords:** oxygen evolution reaction, Cu substitution, spinel structure, oxygen vacancy

## Abstract

In order to improve the electrochemical performance of the NiCo_2_O_4_ material, Ni ions were partially substituted with Cu^2+^ ions having excellent reducing ability. All of the electrodes were fabricated by growing the Ni_1−x_Cu_x_Co_2_O_4_ electrode spinel-structural active materials onto the graphite felt (GF). Five types of electrodes, NiCo_2_O_4_/GF, Ni_0.875_Cu_0.125_Co_2_O_4_/GF, Ni_0.75_Cu_0.25_Co_2_O_4_/GF, Ni_0.625_Cu_0.375_Co_2_O_4_/GF, and Ni_0.5_Cu_0.5_Co_2_O_4_/GF, were prepared for application to the oxygen evolution reaction (OER). As Cu^2+^ ions were substituted, the electrochemical performances of the NiCo_2_O_4_-based structures were improved, and eventually the OER activities were also greatly increased. In particular, the Ni_0.75_Cu_0.25_Co_2_O_4_/GF electrode exhibited the best OER activity in a 1.0 M KOH alkaline electrolyte: the cell voltage required to reach a current density of 10 mA cm^−2^ was only 1.74 V (η = 509 mV), and a low Tafel slope of 119 mV dec^−1^ was obtained. X-ray photoelectron spectroscopy (XPS) analysis of Ni_1−x_Cu_x_Co_2_O_4_/GF before and after OER revealed that oxygen vacancies are formed around active metals by the insertion of Cu ions, which act as OH-adsorption sites, resulting in high OER activity. Additionally, the stability of the Ni_0.75_Cu_0.25_Co_2_O_4_/GF electrode was demonstrated through 1000th repeated OER acceleration stability tests with a high faradaic efficiency of 94.3%.

## 1. Introduction

With the increase in environmental pollution due to excessive use of fossil fuels, studies on new energy conversion and storage systems such as water decomposition devices, fuel cells, and batteries are actively being conducted. In particular, hydrogen energy produced by water splitting is an ideal clean energy and has the advantage of having the largest energy efficiency per unit mass compared to other energy sources [1]. In general, electrolysis of water includes a hydrogen evolution reaction (HER) and an oxygen evolution reaction (OER). In particular, the OER process requires high over-potential, which negatively affects the overall reaction process [2]. It is known that noble metal materials can reduce the over-potential, and in fact, noble metals such as Ir and Ru are already used as efficient electrode active materials [3,4]. However, these noble metals are limited to commercialization on a large scale due to their high price and low reserves, so it is urgent to develop an OER electrode active material with low cost, high efficiency, and high stability for efficient hydrogen production. Recently, Co-based bimetal oxides having a spinel structure as a non-noble metal active material have attracted wide attention in the field of energy storage and conversion.

As an electrode active material for OER, many transition metal compounds having various ligands such as oxide, hydroxide, and phosphide are used [5,6,7], of which Co-based metal oxide is the most promising OER electrode active material available in alkaline media. Moreover, doping a metal ion such as Ni or Fe with a Co-based oxide can further improve electrocatalytic performance. Among them, NiCo_2_O_4_ has high electrical conductivity and stability, attracting much attention from scientists, and is known as a reliable OER electrode active material with excellent repeatability [8]. The general structural formula of a unique spinel structure such as NiCo_2_O_4_ is ACo_2_O_4_, where A is a divalent metal ion such as Ni, Fe, Mn, Mg and Zn, and a cubic packing structure connected to O, where Co^3+^ and A^2+^ ions are respectively the centers of octahedral and tetrahedral [9]. Nickel-cobalt bimetal oxide (NiCo_2_O_4_) is a material that has been studied extensively for a long time and shows ideal electrochemical performance in super capacitors or batteries such as LiB [10], Zn-Air [11], and Li-Air [12]. Recently, NiCo_2_O_4_ has been quite favored in OER, HER, ORR(Oxygen Reduction Reaction), and alcohol oxidation reactions by several researchers [13,14,15,16]. It has been found so far that the electrode potential required to drive a current density of 10 mA cm^−2^ in a NiCo_2_O_4_ electrode active material loaded on a Ni foam support is about 360 mV [17]. Although many researchers are changing the synthetic method and the support to change the high conductivity and active surface area, it has been found that the intrinsic activity of the NiCo_2_O_4_ electrode active material has not improved significantly.

In this study, in order to improve the electrochemical performance of the NiCo_2_O_4_ electrode active material, Ni ions were partially substituted with Cu ions having excellent reducing ability. In 1999, Tavares and his colleagues concluded that the substitution of Cu(II) in the NiCo_2_O_4_ spinel lattice leads to significant changes in surface composition and oxygen evolution [18]. Meanwhile, Mugheri et al. also reported OER performance on NiCo_2_O_4_/CuO electrode active material: 0.5 g of CuO on NiCo_2_O_4_ showed a low starting potential of 1.46 V, and a current density of 10 mA cm^−2^ was achieved at an electrode potential of 230 mV, and the durability was maintained for 35 h [19]. On the other hand, due to the similar physical properties of Ni and Co, the preparation of nickel-cobalt bimetal oxide of NiCo_2_O_4_ is relatively simple and a pure phase structure is easily formed. Synthesis methods vary from sol–gel, chemical precipitation, spray pyrolysis, and electrospinning methods, but many researchers have been the solvothermal or hydrothermal synthesis methods, which are a relatively efficient method to produce NiCo_2_O_4_ nanomaterials [20,21]. In addition, nanoparticles of NiCo_2_O_4_ have various morphologies such as nanowires, nanorods, nanosheets, nanoparticles, hollow nanospheres, and 3-D nanoflowers, and the electrochemical properties vary depending on the morphologies [22,23]. In our study, the graphite felt (GF) electrode support was hydrothermally treated with metal precursors of electrode active materials in synthetic solution. An electrode made by stably growing NiCo_2_O_4_ or Ni_1−x_Cu_x_Co_2_O_4_ nanoparticles on a GF support was used as a water electrolysis catalyst. Moreover, instead of Ni in the NiCo_2_O_4_ spinel structure, Cu was partially substituted, the optimal amount was confirmed, and the OER performance was checked to investigate their correlation.

## 2. Materials and Methods

### 2.1. Fabrications of NiCo_2_O_4_/GF and Ni_1−x_Cu_x_Co_2_O_4_/GF Electrodes

**Scheme 1:**
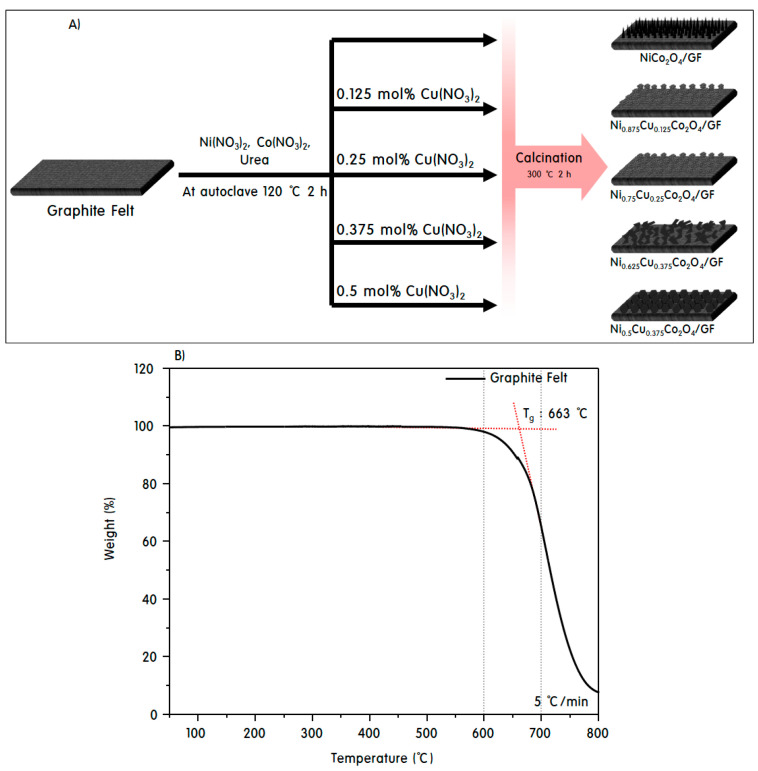
(**A**) Synthesis of Ni_1−x_Cu_x_Co_2_O_4_/graphite felt (GF). (**B**) Graphite felt air atmosphere TGA analysis.

OER electrodes are manufactured by growing active material nanoparticles on the surface of the support electrode by the hydrothermal method as shown in Scheme 1A GF030 (Graphite Felt (GF), 3 mm, CeTech Co., Wuri Dist., Taichung City, Taiwan) was used as an electrode support, and was cut to a width × length × thickness of 10 mm × 10 mm × 1.5 mm. As shown in Scheme 1B, the GF was thermally stabilized by heating at 800 °C at a rate of 5 °C min^−1^ using a thermogravimetric analyzer (TGA, TGA-N1000, Sinco, Daejeon, Korea) under air atmosphere. As a result, it was confirmed that there was almost no decrease in mass to a temperature of 500 °C [24]. Before the nanoparticles were coated, in order to remove the oil film or organic impurities on the surface of the electrode support, it was heated up to 450 °C at a rate of 10 °C min^−1^ in an electric furnace and held at 450 °C for 3 h. After cooling, the surface was etched by ultrasonic treatment for 30 min in a strong acid solution to facilitate coating of the electrode active material. After washing with deionized water and ethanol several times, it was dried in a vacuum oven at 110 °C for 6 h.

First, the hydrothermally fabrication method of the NiCo_2_O_4_/GF electrode is as follows: 10 mmol of Ni(NO_3_)_2_∙6H_2_O (≥98%, Junsei, Tokyo, Japan) and 20 mmol of Co(NO_3_)_2_∙6H_2_O (≥98%, Junsei, Tokyo, Japan) were dissolved in 50 mL distilled water for 1 h. Into the uniformly mixed solution, 0.2 mol of urea (≥98%, Junsei, Tokyo, Japan) was added and stirred for 2 h at room temperature. The GF support electrode was put into this solution, and was sonicated for 6 h to allow the solution to sufficiently penetrate into the support electrode. The final solution is transferred to an autoclave, and the temperature is raised to 120 °C at a heating rate of 5 °C min^−1^ and maintained for 3 h. After the reaction is completed, it is cooled to room temperature and opened to confirm that the electrode active material is coated on the support electrode. It was confirmed that the surface of the obtained electrode was coated with a light purple color, which was washed several times with water and ethanol and then dried at 70 °C for 12 h. Finally, the NiCo_2_O_4_/GF electrode fabrication is completed through the calcination step at 350 °C for 3 h in an air atmosphere.

In the next step, Ni_1−x_Cu_x_Co_2_O_4_/GF (x = 0.125, 0.25, 0.375, and 0.5) electrodes were also made in the same way as NiCo_2_O_4_/GF electrode fabrication. Of Ni(NO_3_)_2_∙6H_2_O 8.75, 7.50, 6.25, and 5.00 mmol, 1.25, 2.50, 6.25, and 5.00 mmol of Cu(NO_3_)_2_∙6H_2_O (≥98%, Junsei, Tokyo, Japan), and 0.02 mol of Co(NO_3_)_2_∙6H_2_O were dissolved in 50 mL of distilled water and stirred for 1 h. Of urea 0.2 mol was added to the uniformly mixed solutions and stirred for 2 h at room temperature. Here, the GF support electrodes were put in the solutions, and those were ultrasonic treated for 6 h. The final solutions containing GF support electrodes were transferred to autoclaves, heated to 120 °C at a rate of 5 °C min^−1^ and maintained for 3 h. After completion of the reaction, the mixtures were cooled to room temperature, and it was confirmed that the electrode active materials were well loaded on the support electrodes. It was convinced that the electrode active materials were coated on the support electrode surfaces, since the electrode surfaces turned from light purple to light brown. In particular, the brown colors of the electrode surfaces were noticeable according to the content of Cu. The electrodes were washed several times with water and ethanol, and then dried at 70 °C for 12 h. Finally, a Ni_1−x_Cu_x_Co_2_O_4_/GF electrodes were obtained through calcination processes at 350 °C for 3 h in air atmospheres. The amount of active materials loaded on the GF support electrode was 10 mg, which was constant for all electrodes.

### 2.2. Evaluation of Physicochemical Properties of Electrodes

Crystal structures of the Ni_1−x_Cu_x_Co_2_O_4_ active material grown on the GF support electrode were confirmed by XRD (Miniflex, Rigaku, Tokyo, Japan) using nickel-filtered Cu *K*α radiation (30 kV, 15 mA) in the 2θ range of 20–90°. The surface morphologies of these active materials were observed with a Hitachi S-4100 field emission scanning electron microscope (SEM). Energy dispersive X-ray spectroscopy (EDS) and EDS elemental mapping were performed by EDAX (EX-250, Horiba, Tokyo, Japan) to know the composition of the elements constituting the Ni_1−x_Cu_x_Co_2_O_4_ active materials. The determinant analysis and SAED pattern for the best performing active material were investigated with a high-resolution transmission electron microscopy (HR-TEM, FEI’s Titan G2 STEM, FEI Company, Hillsboro, Oregon, USA) instrument. The oxidation states of the elements present in the electrode active materials before and after the OER reaction were confirmed using X-ray photoelectron spectroscopy (XPS, K-Alpha Compact XPS, Al *K*α 1486.6 eV, Thermo Scientific, Waltham, MA, USA).

### 2.3. Electrochemical Characterizations of Electrodes

All electrochemical measurements were performed with an electrochemical cell test system (IVIUMnSTAT, Ivium technologies, Eindhoven, Netherlands) at room temperature of 25 °C. The electrochemical OER activity was performed using a standard 3-electrode type electrochemical device, and was performed in an alkaline electrolyte (1.0 M KOH, pH = 14). Here, Ni_1−x_Cu_x_Co_2_O_4_/GF as a working electrode, Hg/Hg_2_Cl_2_ (saturated caramel electrode, SCE) as a reference electrode, and a Pt wire having a thickness of 0.2 mm as a counter electrode were used, respectively. Linear sweep voltammetry (LSV) measurements were performed at a scan rate of 5 mV s^−1^ at 1.0 M KOH (pH = 14). Electrochemical impedance spectroscopy (EIS) measurements were made by applying an amplitude range of 5 mV at frequencies ranging from 100 kHz to 0.01 Hz. Electrochemically active surface area (ECSA) measurements were performed at varying scan speeds from 10 to 100 mV s^−1^ in the 0.967–1.067 V_RHE_ range. The long-term stability test was confirmed by measuring the change in voltage by applying a current of 10 mA cm^−2^. To calculate the Faradaic efficiency, a closed reactor was constructed and used for the OER reaction, and the gases inside the reactor were sampled for 4 h at 30 min intervals using a microsyringe. The sampled gases were injected into gas chromatography (GC, Master GC, Scinco, Daejeon, Korea) equipped with a thermal conductivity detector (TCD), and the amount of oxygen gas generated after the OER reaction was analyzed. The measured potential was converted to reversible hydrogen electrode potential (VRHE) using the following calibration equation [25].

V_RHE_ = V_SCE_ + 0.241 + 0.059 × pH (1)

## 3. Results and Discussion

### 3.1. Physicochemical Properties of Ni_1−x_Cu_x_Co_2_O_4_ Electrode Active Materials Grown on GF

**Figure 1:**
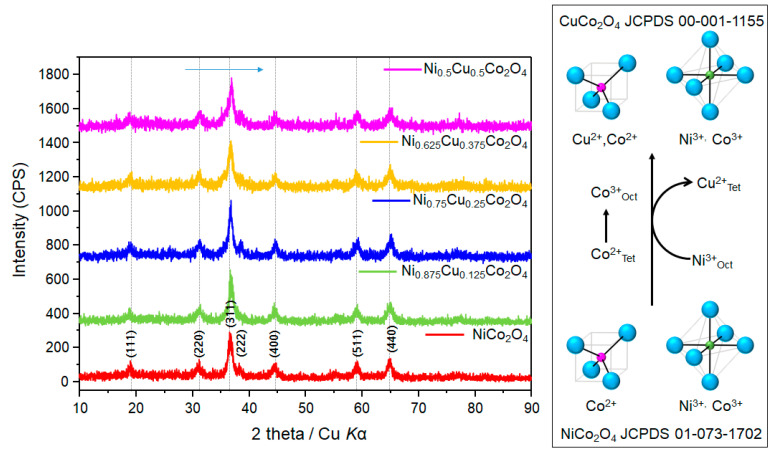
XRD pattern of synthesized Ni_1−x_Cu_x_Co_2_O_4_ active materials.

XRD patterns of Ni_1−x_Cu_x_Co_2_O_4_ electrode active materials grown on the support electrode surface are compared in Figure 1. NiCo_2_O_4_ was consistent with the cubic spinel structure (JCPDS card no. 01-073-1702) with a space group of Fd-3m, peaks corresponding to (111), (220), (311), and (400) facets were observed at 2-theta = 18.9°, 31.2°, 36.7°, and 44.6° [26]. NiCo_2_O_4_ is a p-type semiconductor having a band gap of 2.1 eV, and is a ferrimagnetic material having a number of oxidation–reduction states and excellent electrical conductivity. Ni cations occupy octahedral sites, whereas Co cations coexist in tetrahedral and octahedral sites, so they can be expressed in the form of [Co^2+^][Ni^3+^Co^3+^]O_4_ [27]. In a conventional unit cell of the AB_2_O_4_ spinel structure, O atoms are arranged in a face centered cubic lattice, and there are tetrahedral (8A) sites and octahedral (16B) sites available for the cations. If metal atoms occupy the 8A sites and Co atoms occupy the 16B sites, then the structure is named normal spinel. Conversely, if M atoms occupy half of the 16B sites, while Co atoms occupy the 8A sites and the left half of the 16B sites, then the structure is named inverse spinel. Therefore, it can be said that NiCo_2_O_4_ has a typical inverse spinel structure [28]. Here, if a part of the Ni metal is replaced with Cu^2+^ ions, the Cu^2+^ ions do not occupy the Ni^2+^ sites, but are substituted at the tetrahedral sites of Co^2+^, and the Co ions occupying the tetrahedral sites move to the octahedral sites of the empty Ni^3+^ [29]. In conclusion, Ni_1−x_Cu_x_Co_2_O_4_ crystals in which Ni is partially substituted with Cu can be accurately expressed as (Co^2+^_1−x_Cu^2+^_x_)^tet^(Ni^3+^_1−x_Co^3+^_1+x_)^oct^O_4_. All of Ni_1−x_Cu_x_Co_2_O_4_ materials substituted with Cu^2+^ in the XRD pattern also showed cubic spinel crystals with a space group of Fd-3m. However, these coincide with the peaks at 2-theta = 19.1°, 31.4°, 37.0°, and 45.1° corresponding to (111), (220), (311), and (400) facets of CuCo_2_O_4_ (JCPDS No. 00-001-1155), which are shifted at a higher angle compared to the peaks of the NiCo_2_O_4_ crystals [30]. This is due to the different bond distances and angles between Metal-O due to the different radius of Cu, Co, and Ni ions. According to Bragg’s equation, the shift of the peak at a high angle by substitution of Cu means that it has a smaller d-spacing [31]. Meanwhile, it is well known, the degree of spinel inversion, defined as the proportion of Ni ions on the Oh site, was found to be critical for high conductivity [32]. Therefore, how much the Ni^3+^ occupied at the octahedral site changes due to Cu substitution will be a factor in measuring conductivity.

**Figure 2:**
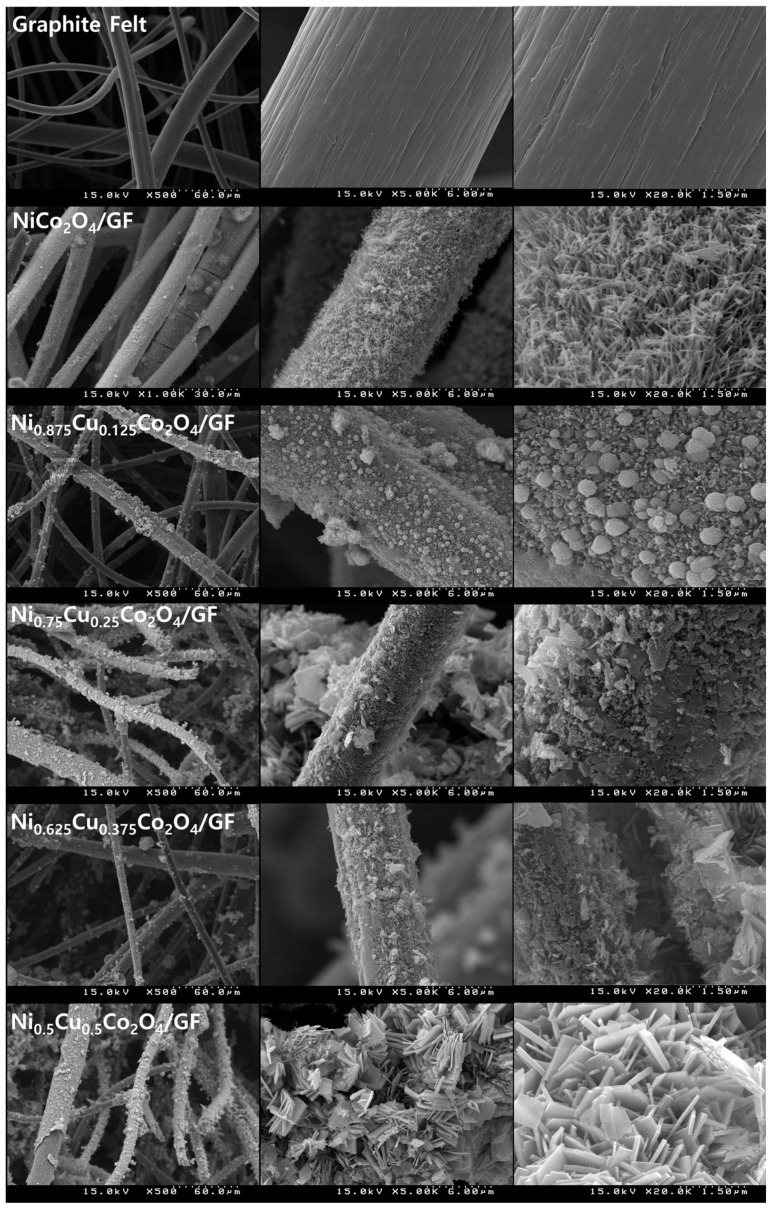
SEM images for the surfaces of Ni_1−x_Cu_x_Co_2_O_4_/GF electrodes.

Figure 2 shows the morphologies of the GF surface and Ni_1−x_Cu_x_Co_2_O_4_ electrode active materials grown on the GF surface. The surface of the pure GF support electrode was smooth overall, and scratches were partially visible on the surface. From this, it was confirmed that the support electrode surface was well etched through heat and acid treatment in the precoating treatment step. On the surface of the NiCo_2_O_4_/GF electrode, small nanorods corresponding to 500 nm in length grew sprawling like dense hairs. However, as Cu was substituted for Ni, the nanorod shaped particles disappeared and the particles with angular plate appeared. The rod and plate shape were mixed until the substitution amount of Cu was 0.375 mol, and the crystal growth was not high compared to NiCo_2_O_4_. It seems that irregular crystals with various sizes and various particle shapes were grown on the GF surface by partially inhibiting the growth between Ni-O-Co as Cu was substituted at the Ni site during the crystal growth process. As a result, it is believed that the irregularity of the crystal shape can lower the activation energy of the reaction by increasing the surface area and the number of reaction active sites [33]. However, in the Ni_0.5_Cu_0.5_Co_2_O_4_/GF electrode where Cu and Ni were added 1:1, regular Ni_0.5_Cu_0.5_Co_2_O_4_ particles with a square plate with a thickness of 70–150 nm and a width of 700 nm were grown on the GF support electrode. It has been confirmed that these largely grown particles are expected to be a factor to lower the electrode performance by increasing the surface resistance.

**Figure 3:**
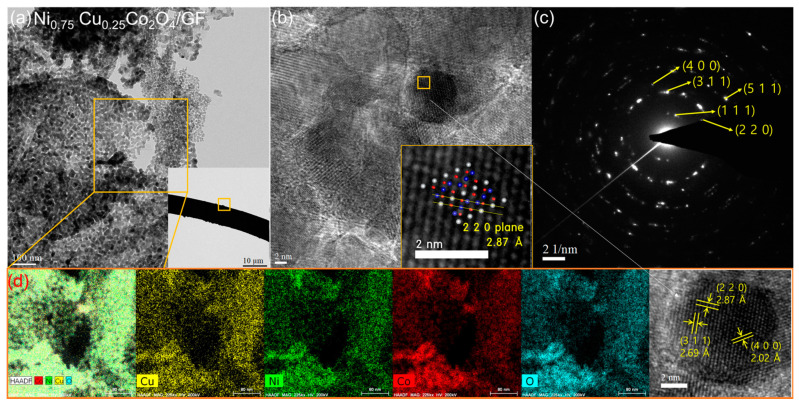
TEM image of Ni_0.75_Cu_0.25_Co_2_O_4_/GF electrode (**a**), HR-TEM images of the electrode surface (**b**), SAED patterns (**c**), and STEM-EDS mapping for all elements (**d**).

Figure 3 shows the HR-TEM image, lattice fringe, SAED pattern, and element mapping image, which measured and dispersing it in ethanol solvent after scraping off the Ni_0.75_Cu_0.25_Co_2_O_4_ electrode active materials grown on the support electrode surface. Figure 3a shows the image of the dispersed Ni_0.75_Cu_0.25_Co_2_O_4_ particles. The particle size could not be confirmed clearly in the SEM photograph of Figure 2, but in the HR-TEM photograph, it was confirmed that the Ni_0.75_Cu_0.25_Co_2_O_4_ was composed of very small nanocrystals, and the size was about 10–20 nm. Figure 3b shows the lattice fringes in the selected area. The most clearly visible grating is a (220) facet having an interplane distance of 2.87 Å, a characteristic surface of the spinel structure [34]. Figure 3c shows the SAED (selected area diffraction) pattern at the same point as Figure 3a. The reciprocal of the distance from the center to the spot corresponds to the distance between the lattices, and (111), (220), (311), (400), and (511) facets were identified. It was found that Ni_0.75_Cu_0.25_Co_2_O_4_ particles are polycrystals in which various single crystals are mixed in various orientations [35]. In Figure 3d, the composed elementals in Ni_0.75_Cu_0.25_Co_2_O_4_ particle were confirmed by the STEM-element mapping image analysis, and the intensity of the color is proportional to the element concentration. Elements were evenly distributed on the particles, and no entangled elements were visible. Table 1 shows the atomic composition of the elements present in the assembled electrodes. The component concentrations present in most of the electrodes were almost stoichiometrically similar to the amount added during the preparing process. In particular, the atomic ratio of the constituent elements was Ni:Cu:Co:O = 10.3(3.1):3.2(1.0):28.0(8.8):59.9(18.7), which was almost quantitatively consistent with the molar ratio of the atoms (Ni:Cu:Co:O = 3:1:8:16) of the synthesized Ni_0.75_Cu_0.25_Co_2_O_4_ particles.

### 3.2. Electrochemical Properties of Ni_1−x_Cu_x_Co_2_O_4_ Electrode Active Materials Grown on GF

**Figure 4:**
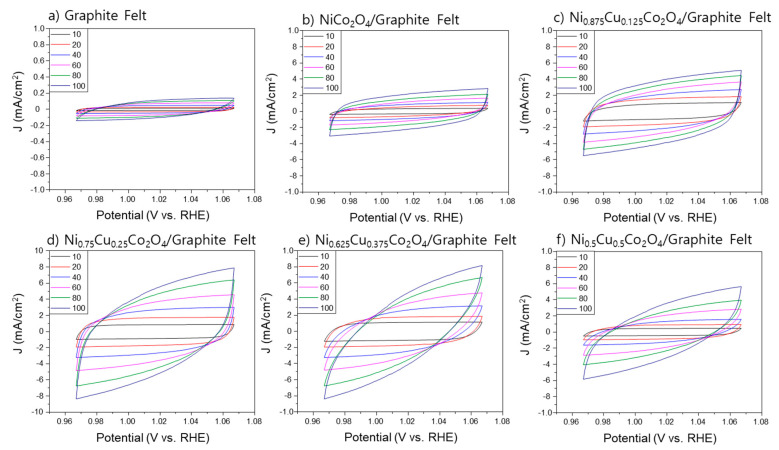
Cyclic voltammetry with scan rates in the non-faradaic region for all electrodes.

Among the properties of catalysts used for electrochemical water decomposition, one of the important factors is the high electrochemically active surface area (ECSA) [36]. To predict ECSA, cyclic voltammetry (CV) was measured by varying the scan rate in the potential range of the non-faradaic region, and the current densities as a function of potential are plotted in Figure 4. In general, the rectangular shape of CV curve mean electrolyte ions accumulated on the electrode surface (electric double layer formation), indicating pure capacitive energy storage. When the redox current density is large in the same voltage range, the electroactive capacity is also large [37]. As the CV cycle increased, the redox current density in all samples increased. The redox current density increased as Cu was substituted, and it was found that the current density of Ni_0.75_Cu_0.25_Co_2_O_4_ increased drastically when the redox reactions occurred.

**Figure 5:**
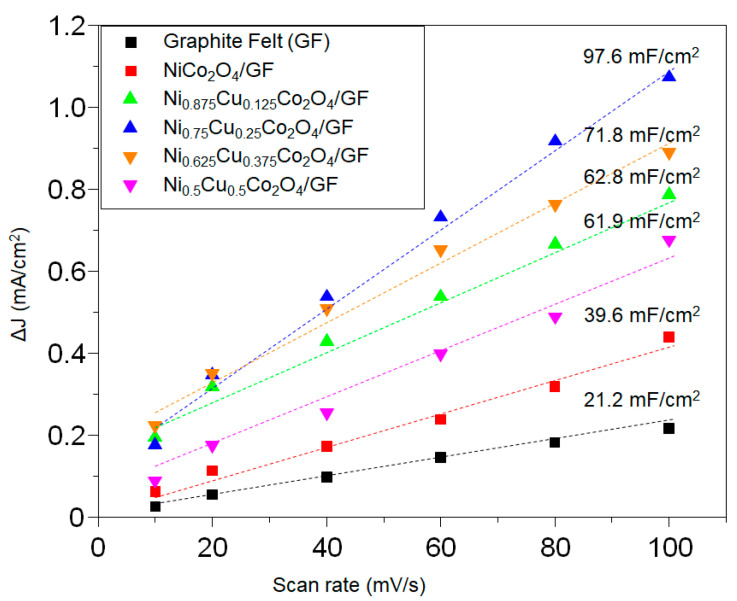
Current density differences depending on scan rate for all electrodes.

The current density differences (Janodic–Jcathodic, ΔJ) in all of electrodes are shown in Figure 5. The linear trend line slope was plotted with the values obtained in Figure 4 to measure the double layer capacitance (C_dl_, double-layer capacitance, ESCA) and compared. The C_dl_ of GF calculated from the slope was calculated to be 21.2 mF cm^−2^, and the C_dl_ value increased as a whole in the electrode active material coated on the GF surface. Especially for Ni_0.75_Cu_0.25_Co_2_O_4_/GF showed the highest ECSA value of 97.6 mF cm^−2^, compared to the electrodes of NiCo_2_O_4_/GF (39.6 mF cm^−2^), Ni_0.875_Cu_0.125_Co_2_O_4_/GF (62.8 mF cm^−2^), Ni_0.625_Cu_0.375_Co_2_O_4_/GF (71.8 mF cm^−2^), and Ni_0.5_Cu_0.5_Co_2_O_4_/GF (61.9 mF cm^−2^). From these results, we predicted that Ni_0.75_Cu_0.25_Co_2_O_4_/GF will show a higher catalytically active site and will show excellent electrochemical catalyst performance [38].

**Figure 6:**
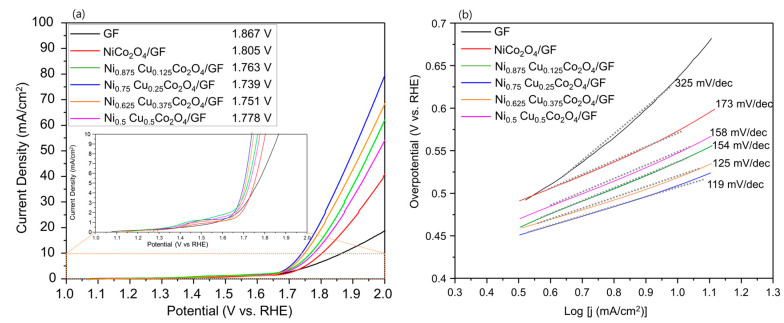
Linear scan voltammetry (LSV) curves for oxygen evolution reaction (OER) polarization of Ni_1−x_Cu_x_Co_2_O_4_/GF (**a**) and Tafel plots (**b**).

To compare the OER activity of the electrodes, LSV (linear scan voltammetry) curves were measured for all electrodes, and the results are shown in Figure 6a. In general, the electrolytic reaction describes the OER reactivity of the electrode by comparing the electrode potential at a current density of 10 mA cm^−2^ [39]. In order to reach a current density of 10 mA cm^−2^ at the GF support electrode, an overvoltage of 1.87 V is required. In Ni_1−x_Cu_x_Co_2_O_4_/GF electrodes, electrode potentials were obtained with 1.81 (η = 575 mV), 1.76 (η = 533 mV), 1.74 (η = 509 mV), 1.75 (η = 521 mV), and 1.79 V (η = 548 mV) in NiCo_2_O_4_/GF, Ni_0.875_Cu_0.125_Co_2_O_4_/GF, Ni_0.75_Cu_0.25_Co_2_O_4_/GF, Ni_0.625_Cu_0.375_Co_2_O_4_/GF, and Ni_0.5_Cu_0.5_Co_2_O_4_/GF electrodes, respectively. In particular, the Ni_0.75_Cu_0.25_Co_2_O_4_/GF electrode showed the lowest electrode potential, which means that it shows the best OER activity. Figure 6b shows the Tafel slope value derived from the LSV curve, and the Tafel slope shows the unique properties of the electrochemical catalytic electrode. The Tafel slope is the value of the electrode potential required when 10-fold electrons flow, and it is reported that the lower this value, the better the water-oxidation kinetics [40]. In Figure 6b, the Tafel slope of GF showed 325 mV dec^−1^, and the Ni_0.75_Cu_0.25_Co_2_O_4_/GF electrode, which expressed the lowest electrode potential in the LSV curve, also showed the lowest Tafel slope with 119 mV dec^−1^. For the NiCo_2_O_4_/GF electrode 173 mV dec^−1^, 154 mV dec^−1^ for the Ni_0.875_Cu_0.125_Co_2_O_4_/GF electrode, 125 mV dec^−1^ for Ni_0.625_Cu_0.375_Co_2_O_4_/GF electrode, and 158 mV dec^−1^ for Ni_0.5_Cu_0.5_Co_2_O_4_/GF electrode were respectively obtained. These values were consistent with OER performance. Table 2 summarizes and compares the results of using noble or non-noble metal electrodes to recognize whether the electrochemical performance of Ni_1−x_Cu_x_Co_2_O_4_ electrode is high or low. The overpotential was widely distributed from 241 to 540 mV, and the Tafel values were also varied from 53 to 238 mV dec^−1^. When nickel foam was used as a support electrode, the electrochemical performance was relatively excellent, whereas when a carbon electrode was used, it was significantly reduced. This is because the carbon electrode has a lower conductivity than the metal electrode. In addition, the performances vary depending on the shapes of graphite, but it is confirmed that the electrochemical performance was slightly lower than when using other carbon support electrodes because electrolyte wettability was lowered when GF was used. In the end, the performance in this study using GF was not largely improved in this reason.

**Figure 7:**
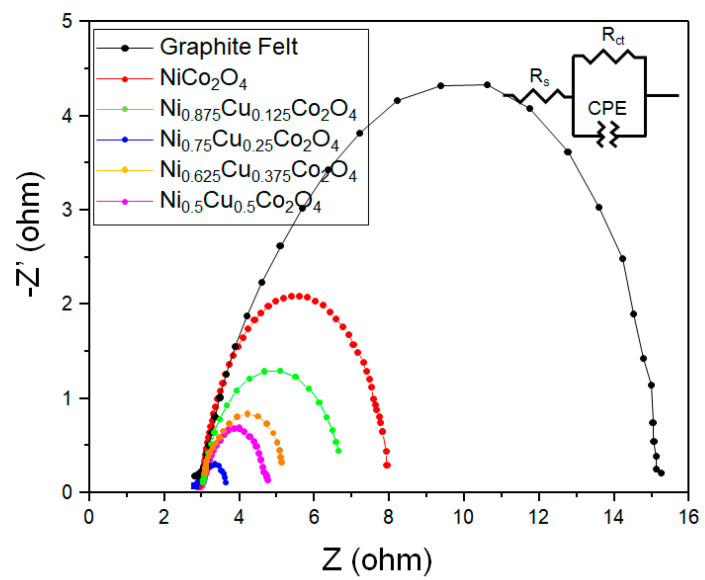
Nyquist plots for all electrodes under an applied potential of 1.7 V.

Electrochemical impedance spectroscopy (EIS) was performed to investigate the charge transfer properties of the electrode, in Figure 7. Generally, the size of the semicircle of the Nyquist plot is assigned to the charge transfer resistance (R_ct_) [51]. Table 3 shows the R_s_ (solution resistance) and R_ct_ values for all of electrodes. The R_ct_ value was found to be 10.39 Ω, which was the highest value in the support electrode GF, and greatly decreased to 1.814 Ω in the NiCo_2_O_4_/GF electrode. Particularly, in the electrode substituted with 0.25 mol of Cu, the size of the electrode active material was reduced, and they were thinly and evenly grown on the electrode surface, and eventually the charge transfer resistance was significantly reduced to 0.728 Ω in the Ni_0.75_Cu_0.25_Co_2_O_4_/GF electrode. However, when Cu was replaced with more than that, the size of the electrode active material increased and the thickness of the plate-like particles thickened. As a result, the active area of the electrode surface decreased, and eventually the charge transfer resistance increased again to 1.631 Ω in the Ni_0.5_Cu_0.5_Co_2_O_4_/GF electrode.

### 3.3. Evaluation of the Durability of the Electrode

**Figure 8:**
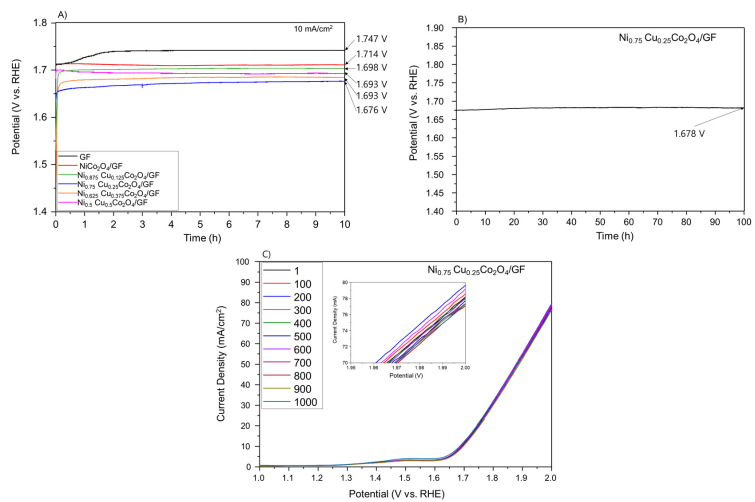
Chronopotentiometry of all electrodes for 10 h at 10 mA cm^−2^ (**A**), Chronopotentiometry for 100 h on the Ni_0.75_Cu_0.25_Co_2_O_4_/GF electrode (**B**), and 1000th cycled LSV curves for the Ni_0.75_Cu_0.25_Co_2_O_4_/GF electrode (**C**).

The long-term durability of the electrode serves as an important factor in evaluating the electrochemical material [52]. The stability of all electrodes was evaluated by applying a current of 10 mA cm^−2^ through chronopotentiometry for a total of 10 h, and the results are shown in Figure 8A. In the electrode coated with the electrode active material, the potential was maintained stably over time, but the GF support electrode showed an unstable potential until about 2 h, and it can be seen that the overpotential greatly increased after that. This can be attributed to the oxidization of the surface of the GF by continuous reaction [53]. Figure 8B shows the results for 100 h to further confirm the long-term stability of the Ni_0.75_Cu_0.25_Co_2_O_4_/GF electrode, which showed the highest performance. The initial stable voltage was maintained, and there was no significant difference in potential even after 100 h of reaction. It is judged that in the electrode coated with the electrode active material containing Cu, the OH^−^ intermediate formed on the electrode surface was rapidly oxidized by oxygen to suppress oxidation of the electrode surface, and as a result, a stable performance could be maintained [54]. Continuous accelerated stability testing is an important factor in evaluating the durability of the OER. Thus, the LSV curves for 1000 cycles at the Ni_0.75_Cu_0.25_Co_2_O_4_/GF electrode are presented in Figure 8C. Up to 300 cycles, the OER performance was rather improved, and gradually decreased from 400 cycles, but there was little change in performance from 700 cycles. This result means that the partial introduction of Cu into the lattice of NiCo_2_O_4_ not only improves the electrochemical performance of NiCo_2_O_4_, but also has a pretty positive effect on stability.

In order to know the difference between the actual efficiency and the theoretical efficiency of the Ni_0.75_Cu_0.25_Co_2_O_4_/GF electrode, an OER electrolysis reaction was performed while applying a current of 10 mA cm^−2^ for 4 h. At this time, the amount of oxygen generated was measured, and the theoretically calculated amount was compared with the actually generated amount. The theoretically calculated amount of oxygen was calculated using the following equation from Faraday’s law [55].

(2) $$n_{O_{2}}\left(\mathrm{theoretical}\right)=\frac{Q}{nF}=\frac{I\times t}{nF}=\frac{0.01\;A\;\times 14400\;s}{4\times 96485.3\;s\cdot A\cdot {\mathrm{mol}}^{-1}}=3.73\times 10^{-4}\;\mathrm{mol}$$

where $n_{O_{2}}$ is the theoretically calculated amount of O_2_, Q is the amount of applied charge, *n* is the number of electrons participating in the OER reaction (4 electrons), *F* is the Faraday constant (96485.3 s A mol^−1^), *i* is the applied current (0.01 A), and *t* is the reaction time (14,400 s). Faraday efficiency is calculated using the following equation.

(3) $$Faradaic\;efficiency=\;\frac{n_{O_{2}}\left(\mathrm{measured}\right)}{n_{O_{2}}\left(\mathrm{theoretical}\right)}$$

**Figure 9:**
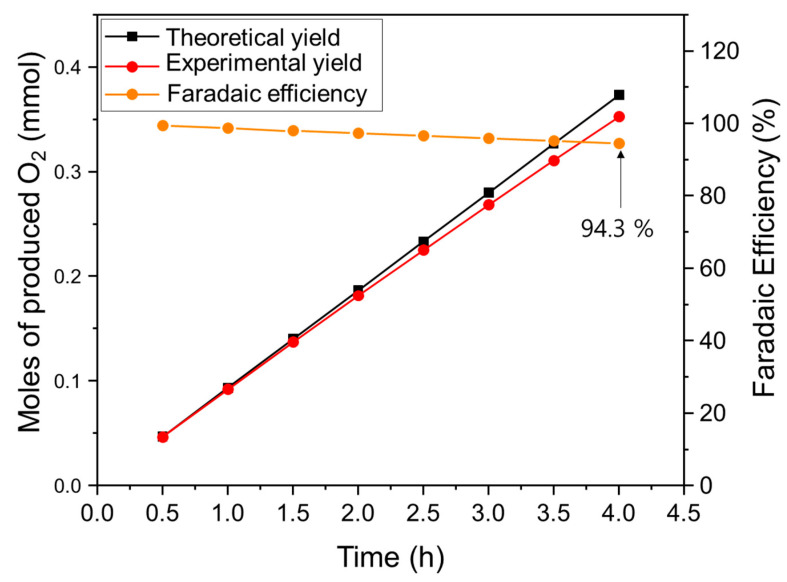
Faradaic efficiency of Ni_0.75_Cu_0.25_Co_2_O_4_/GF for the theoretically calculated and experimentally measured O_2_ at a current density of 10 mA cm^−2^.

As a result of calculation, the Faraday efficiency of about 94.3% was exhibited at the Ni_0.75_Cu_0.25_Co_2_O_4_/GF electrode as shown in Figure 9, and this value means that almost all of the applied electrons were used for the oxygenation reaction without other side reactions except for experimental error.

**Figure 10:**
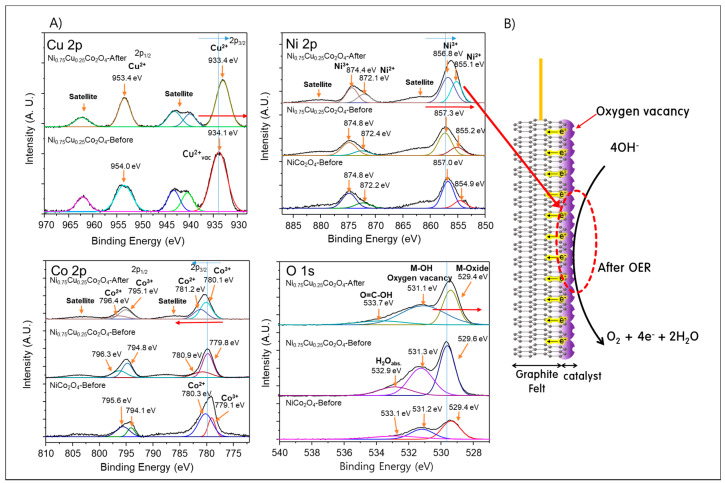
XPS spectra for the NiCo_2_O_4_ electrode (**A**) and surface phenomena on the Ni_0.75_Cu_0.25_Co_2_O_4_/GF electrode before and after the OER reaction (**B**).

In order to confirm the change in the oxidation state of the active elements after the 1000th OER cycle in the Ni_0.75_Cu_0.25_Co_2_O_4_/GF electrode showing the best performance, Figure 10A shows the XPS results before and after the OER reaction in the Ni_0.75_Cu_0.25_Co_2_O_4_/GF electrode. In addition, the XPS result before the OER reaction in the NiCo_2_O_4_/GF electrode was compared with those. At the Ni_0.75_Cu_0.25_Co_2_O_4_/GF electrode before the OER reaction, peaks which corresponding to 2p_3/2_ and 2p_1/2_ electron spins of Cu^2+^ were identified at 934.1 eV and 954.0 eV, respectively [56]. The difference in the separated binding energy was 19.9 eV, similar to the value reported in the literature, 19.8 eV [57]. In addition, a satellite peak was appeared at 940–943 eV, which is a peak caused by the transfer of 3d electrons (d–d transfer) between the metal and the ligand, and is a major factor in confirming the presence of Cu^2+^ [58]. In the electrode after the reaction, the peaks of Cu 2p_3/2_ and Cu 2p_1/2_ shifted to 933.4 eV and 953.4 eV, respectively. We were convinced from the slight decrease in the binding energy after the 1000th OER reaction that Cu^2+^ ions were attracted the electrons, which emitted by oxidation of OH^−^ ions during the OER reaction. This is probably evidence that the evolution of oxygen in the spinel lattice occurs at the oxygen site linked to Cu^2+^. That is, as Cu^2+^, which has a higher potential than Ni^2+^ and Co^2+^, is spontaneously reduced, oxygen vacancies are formed around it, and the vacancies easily adsorbed water or OH^−^, which in turn improves OER performance. In the Ni 2p XPS spectrum of the NiCo_2_O_4_/GF electrode, peaks that correspond to Ni^2+^ 2p_3/2_, Ni^3+^ 2p_3/2_, Ni^2+^ 2p_1/2_, and Ni^3+^ 2p_1/2_ appeared respectively at the binding energies of 854.9, 857.0, 872.2, and 874.8 eV [59]. In the Ni 2p XPS spectra of the Cu-containing Ni_0.75_Cu_0.25_Co_2_O_4_/GF electrode, they were found at 855.2, 857.3, 872.4, and 874.8 eV, respectively. As Cu was introduced, the binding energy of Ni moved to the slightly higher (Ni^3+^). This is because some of the Ni^2+^ present in the tetrahedral site moves to the octahedral site by the introduction of Cu and forms a stronger bond with oxygen [60]. In the spectrum of the Ni_0.75_Cu_0.25_Co_2_O_4_/GF electrode after the OER reaction, peaks were observed at 855.1, 856.8, 872.1, and 874.4 eV, respectively. This is a decrease of about 0.1–0.4 eV from the binding energy before the reaction. Moreover, the area of the peak of Ni^2+^ increased significantly compared to that before the reaction, which means that Ni^3+^ (maybe NiOOH) is being reduced to Ni^2+^ during the OER reaction. In the Co 2p XPS spectrum of NiCo_2_O_4_/GF, peaks corresponding to Co^3+^ 2p_3/2_, Co^2+^ 2p_3/2_, Co^3+^ 2p_1/2_, and Co^2+^ 2p_1/2_ were identified at 779.1, 780.3, 794.1, and 795.6 eV, respectively. From this result, we were convinced that Co^3+^ and Co^2+^ coexisted in this sample [61]. As Cu^2+^ was introduced, Co^2+^ present in the Td site was oxidized to Co^3+^ while moving to the Oh site, so the proportion of Co^3+^ increased [62]. Therefore, the peaks at the Ni_0.75_Cu_0.25_Co_2_O_4_/GF electrode before the reaction appeared at 779.8, 780.9, 794.8, and 796.3 eV, and these shifted to a higher binding energy of about 0.3–0.7 eV compared to the NiCo_2_O_4_/GF electrode. In the Ni_0.75_Cu_0.25_Co_2_O_4_/GF electrode after the reaction, these peaks shifted to slightly higher binding energies of 780.1, 781.2, 795.1, and 796.4 eV, respectively. However, the shift was too small. This means that CoOOH was changed to Co_2_O_3_ rather than a change from Co_2_O_3_ to CoO during the OER reaction. For O 1s in the NiCo_2_O_4_/GF electrode, peaks corresponding to M-O, oxygen defects and M-OH, and physically adsorbed H_2_O were identified at 529.4, 531.2, and 533.1 eV, respectively [63]. As Cu was introduced, the peak areas for M-O and M-OH were greatly increased. This means that oxygen defects have already occurred in the spinel lattice due to the introduction of copper before the OER reaction. In the Ni_0.75_Cu_0.25_Co_2_O_4_/GF electrode after the OER reaction, the area of peaks for M-OH and C=O-OH increased over a wide range. This is predictable evidence that oxygen vacancies are formed in the lattice due to the introduction of Cu, and that OH^−^ is easily adsorbed to the defect sites. Figure 10B shows the overall reaction cycle of the OER reaction occurring on the electrode surface. From the stepwise reactions occurring at the interface between the active material of the electrode and the electrolyte during the OER reaction, we can recognize a series of processes in which O_2_ and electrons are generated as the 4OH^−^ ions in the electrolyte are oxidized. In addition, through this cycle, it was confirmed that the oxidation–reduction processes of active metals were consistent with XPS results.

**Scheme 2:**
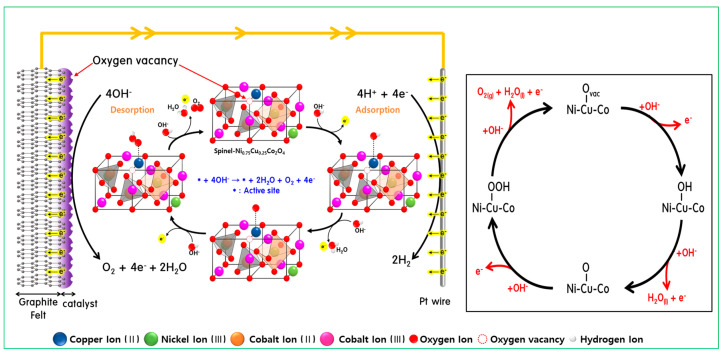
Mechanism of OER on the surface Ni_0.75_Cu_0.25_Co_2_O_4_/GF electrode in alkaline solution.

Based on the above results, the mechanism of the predictable OER reaction was expressed in Scheme 2, and the OER mechanism occurring in the basic solution is simplified as follows [64].

M + OH^−^ → M-OH (4)

MOH + OH^−^ → MO + H_2_O (l) (5)

2MO → 2M + O_2_(g) (6)

MO + OH^−^ → MOOH + e^−^ (7)

MOOH + OH^−^ → M + O_2_(g) + H_2_O (l) (8)

Overall reaction: M + 4OH^−^ → M + O_2_ (g) + 2H_2_O + 4e^−^ (9)

First, with the introduction of Cu^2+^, oxygen vacancies were formed in the Ni_1−x_Cu_x_Co_2_O_4_ (or Ni-Cu-Co-O_vac_) spinel structure due to the relative redox potential difference between Cu^2+^, Ni^2+^, and Co^2+^. In the first step reaction, OH^−^ ions in the electrolyte were adsorbed to oxygen vacancies formed on the surface of the material, generating one electron, and the Ni-Cu-Co-O_vac_ surface becomes (Ni-Cu-Co)-OH. In the second step, H_2_O was generated while another OH^−^ ion of the electrolyte removed H^+^ ions bound to the material, and one electron was generated and the material surface became (Ni-Cu-Co)-O. In the third step, another OH^−^ ion of the electrolyte was bonded to O on the material surface to become (Ni-Cu-Co)-OOH, and one electron was generated. Finally, in the fourth step, another OH^−^ ion in the electrolyte removed H^+^ from the -OOH group on the surface of (Ni-Cu-Co)-OOH, resulting in H_2_O, and one electron was generated. At this time, the remaining -OO was also dissociated to O_2_, and the material returned to the initial state of Ni-Cu-Co-O_vac_. The four electrons generated during the OER reaction were transferred to the GF collector layer and then discharged to the HER electrode through an external circuit.

## 4. Conclusions

For an efficient and stable OER reaction, an OER electrode was prepared by growing a Ni_1−x_Cu_x_Co_2_O_4_ electrode active material on a GF support electrode through hydrothermal synthesis. The pure GF support electrode not only had low performance but also tended to be easily oxidized, but through the method of growing the electrode active material on the surface, it was possible to increase the surface active site and increase the electrode stability for a long time. XRD, SEM, and TEM image analysis was confirmed, showing the change in crystallinity and shape as Cu was partially introduced into the NiCo_2_O_4_ spinel structure. As a result of carrying out a water decomposition OER reaction in 1.0 M KOH alkaline electrolyte, the Tafel value of 325 mV dec^−1^ was found in the GF electrode, but was significantly improved to 119 mV dec^−1^ in the Ni_0.75_Cu_0.25_Co_2_O_4_/GF electrode. In addition, the Ni_0.75_Cu_0.25_Co_2_O_4_/GF electrode exhibited a Faraday efficiency of 94.3% and was confirmed to have a high catalytically active surface area of 97.6 mF cm^−2^. Moreover, the activity was maintained without deterioration even after a long time reaction of more than 1000th cycles. In conclusion, in this study, the adsorption of OH^−^ ions was facilitated through oxygen vacancies caused by the introduction of Cu, and it is considered that the performance was maintained even in long-term reactions.

## Figures and Tables

**Table 1:** Atomic compositions on Ni_1−x_Cu_x_Co_2_O_4_/GF electrodes obtained by the EDS analysis.

	Atomic (%)	Ni	Cu	Co	O	C
Sample						
Graphite Felt		-	-	-	10.49	89.51
NiCo_2_O_4_/GF		7.46	-	14.88	29.82	47.84
Ni_0.875_Cu_0.125_Co_2_O_4_/GF		6.70	0.98	15.40	31.00	45.92
Ni_0.75_Cu_0.25_Co_2_O_4_/GF		5.51	1.70	14.98	32.11	45.70
Ni_0.625_Cu_0.375_Co_2_O_4_/GF		4.59	2.74	14.62	29.27	48.78
NiCuCo_2_O_4_/GF		3.93	3.52	14.83	29.64	48.08

**Table 2:** Comparison of electrochemical performances for noble or non-noble metal electrodes.

Sample	Support	Overpotential (*j* = 10 mA cm^−2^)	Tafel Slope (mV dec^−1^)	Ref.
Ni_0.75_Cu_0.25_Co_2_O_4_	Graphite Felt	509 mV	119	This work
Co-P	Graphite Felt	530 mV (*j* = 5 mA cm^−2^)	133	[41]
CuS/CuFeS_2_	Carbon Felt	400 mV	171	[42]
NiCu(I)	Ni foam	252 mV	54	[43]
NiFe_2_O_4_	Ni plate	520 mV	223	[44]
CuFe_2_O_4_	Ni plate	540 mV	238	[44]
Fe-Co(OH)_2_	Glassy Carbon	290 mV	69	[45]
NiCo_2_S_4_/RGO	Glassy Carbon	366 mV	65	[46]
NiCo_2_O_4_/CoN_x_-NMC NMC (Nitrogen doped Mesoporous Carbon)	Glassy Carbon	370 mV	99	[47]
(Co_0.21_Ni_0.25_Cu_0.54_)_3_Se_2_	Glassy Carbon	241 mV	53	[48]
FeCoNi alloy	N-doped graphene	288 mV	57	[49]
RuO_2_	Glassy Carbon	300 mV	54	[49]
IrO_2_	Glassy Carbon	314 mV	-	[50]

**Table 3:** Resistance behavior for all electrodes.

	Sample	Graphite Felt	NiCo_2_O_4_	Ni_0.875_Cu_0.125_Co_2_O_4_	Ni_0.75_Cu_0.25_Co_2_O_4_	Ni_0.625_Cu_0.375_Co_2_O_4_	Ni_0.5_Cu_0.5_Co_2_O_4_
Factor							
R_S_ (Ω) (Solution Resistance)		2.960	2.768	2.627	2.654	2.613	3.012
R_ct_ (Ω) (Charge Transfer Resistance)		10.39	1.814	1.250	0.728	1.613	1.631
Total resistance (Ω)		13.35	4.582	3.877	3.382	4.226	4.643
